# An overview of statistical methods for biomarkers relevant to early clinical development of cancer immunotherapies

**DOI:** 10.3389/fimmu.2024.1351584

**Published:** 2024-08-21

**Authors:** David Dejardin, Anton Kraxner, Emilie Schindler, Nicolas Städler, Marcel Wolbers

**Affiliations:** ^1^ Data Sciences, Product Development, F. Hoffmann-La Roche AG, Basel, Switzerland; ^2^ Roche Pharma Research and Early Development Oncology, F. Hoffmann-La Roche AG, Basel, Switzerland

**Keywords:** biomarkers, statistical methods, validation, immunotherapy, prognostic model, predictive model

## Abstract

Over the last decade, a new paradigm for cancer therapies has emerged which leverages the immune system to act against the tumor. The novel mechanism of action of these immunotherapies has also introduced new challenges to drug development. Biomarkers play a key role in several areas of early clinical development of immunotherapies including the demonstration of mechanism of action, dose finding and dose optimization, mitigation and prevention of adverse reactions, and patient enrichment and indication prioritization. We discuss statistical principles and methods for establishing the prognostic, predictive aspect of a (set of) biomarker and for linking the change in biomarkers to clinical efficacy in the context of early development studies. The methods discussed are meant to avoid bias and produce robust and reproducible conclusions. This review is targeted to drug developers and data scientists interested in the strategic usage and analysis of biomarkers in the context of immunotherapies.

## 1 Introduction

Targeted therapies and cancer immuno-therapies (CIT), including immune checkpoint inhibitors (CPI) have revolutionized the treatment paradigm for a number of cancers, leading to improvements in progression-free and overall survival, and more durable responses [reviewed in: Shahid et al. (1); Esfahani et al. (2); Robert (3); Murciano-Goroff et al. (4)]. Despite these successes, many patients do not or only transiently benefit from such treatments and additional efforts are required to truly unleash the full potential of precision and personalized medicine. Biomarkers play an integral role in this process and can guide and impact clinical research and development and, ultimately, actual patient care [reviewed in: (5–8)].

With emerging and evolving technologies, our understanding of cancer biology and tumor immunology is growing, which also is helping the discovery and development of novel biomarkers. Biomarkers may already play an important role by providing answers to key questions in the following areas of early clinical development of immunotherapies (please also refer to **Table 1** in the supplementary section for examples): 1) Demonstration of mechanism of action (MoA) by analyzing the pharmacodynamic effect. For CIT, characterization of drug effects and efficacy may differ from classical therapies focused on direct tumor cell killing and tumor shrinkage. 2) Dose finding and dose optimization. 3) Mitigation and prevention of adverse reaction to the drug. In particular, new challenges arise from the need to improve tolerability of immunotherapies, and to predict and mitigate adverse immune reactions. 4) Patient enrichment and indication prioritization, typically based on baseline characteristics of the respective tumor such as target expression. Considerations regarding the variety of immune targets leveraged by the new immune therapies, as well as emergence of resistance mechanisms are key to the success of these therapies.

**Table 1:** Biomarker definitions.

Biomarker	A factor that is objectively measured and evaluated as an indicator of normal biological processes, pathogenic processes, or pharmacological responses to a therapeutic intervention (9)
Prognostic biomarker (measured at baseline)	A biomarker used to identify likelihood of a clinical event, disease recurrence or progression in patients who have the disease or medical condition of interest. Prognostic biomarkers are often identified from observational data and are regularly used to identify patients more likely to have a particular outcome.
Predictive biomarker (measured at baseline)	A biomarker used to identify individuals who are more likely than similar individuals without the biomarker to experience a favorable or unfavorable effect from exposure to a medical product or an environmental agent.
Pharmacodynamic biomarker (measured at baseline and on-treatment)	A biomarker that indicates biologic activity of a drug. These biomarkers are usually linked to the MoA but can also be independent of the MoA. These biomarkers may also be related to the clinical activity of the drug.
Safety biomarker (measured at baseline and on-treatment)	A biomarker related to the likelihood, presence, or extent of toxicity as an adverse effect

MoA = Mechanism of Action.

A number of research studies have highlighted challenges and a lack of reproducibility in several areas of biomarker research (10, 11). These challenges are accentuated in the context of immunotherapies due to the complexity of the immune system and the variety of biomarkers studied. A comprehensive review of the statistical principles for biomarkers in CIT is lacking. In this review, we describe these statistical principles focusing to the application of biomarkers in early clinical development studies of CIT. We believe that adherence to these principles improves the quality of biomarker studies and the generalizability and robustness of their findings

The remainder of this manuscript is structured as follows: In Section 2, we review biomarker definitions, examples, and technologies, and typical clinical endpoints. We also connect the previously mentioned four areas of early CIT drug development listed above with the statistical analyses reviewed in the next section. Section 3 describes appropriate statistical methods for biomarkers, starting with the choice of data transformation and probabilistic models (Section 3.1), and relevant biomarker considerations for dose selection and optimization (Section 3.2). Sections 3.3 and 3.4 discuss the assessment of prognostic and predictive characteristics of baseline biomarkers. Section 3.5 covers analysis methods for on-treatment biomarkers including landmarking and joint modeling. Challenges related to high-dimensional biomarker analyses are described in Section 3.6. Finally, Section 3.7 provides examples of the use of PK/PD models in relation to biomarkers. We conclude the manuscript with a brief discussion.

## 2 Biomarker definitions, examples and clinical endpoints

The complexity of the human immune system is reflected in the plethora of biomarkers that have been developed to capture the activation of the immune system against cancer cells, and the interaction between immunotherapies and the immune system. **Supplementary Table 1** in the supplemental material provides examples of biomarkers which have been proposed in the scientific literature. This diversity strengthens the need for a rigorous and robust analysis of the effect of biomarkers.

Biomarkers may serve four different purposes as summarized in **Table 1** and further described below (9, 12).

First, prognostic baseline biomarkers differentiate patients with regards to the outcome of the disease independently of the treatment. A typical example of a prognostic biomarker is the total CD8 count in the tumor. Patients with a high number of CD8+ cells, which identifies the T-cell, have immune response against the tumor and better prognosis (13).

Second, predictive biomarkers at baseline are markers that differentiate patients that benefit most from a treatment. Hence, predictive biomarkers are specific to a (class of) treatments and, sometimes, specific to a tumor type. PD-L1 expression is a predictive biomarker for CPI for some tumor types and disease stages though it has limitations (14).

In clinical trials, prognostic and predictive biomarkers are utilized for the enrichment of the study population to obtain a more homogeneous population. For example, prognostic biomarkers may be used to select only subjects with a poor prognosis who are in most urgent need of better treatment options into the study. Moreover, the power of a trial with a time-to-event endpoint (such as progression- free survival) to detect a targeted hazard ratio depends on the observed number of events and not on the number of recruited subjects (15, chapter 15). In a population with a worse prognosis, these events are observed more quickly allowing for trials with a lower sample size and/or a shorter trial duration. Predictive biomarkers are more specifically utilized to investigate restrictions on the patient population (in the drug label). In a first phase of development, early studies may enroll all-comers. The emerging understanding of cancer biology and the mechanism of action of the drug, supported by data analysis of these early studies may allow definition of a biomarker-positive subgroup with an enhanced treatment effect. In a second phase of development, subsequent trials may restrict the population to patients who benefit most from the drug, based on the identified biomarker or continue with two primary populations: all-comers and the biomarker-positive populations (see section 3.4 for an example), in both cases the biomarker status is determined by a diagnostic test. Moreover, prognostic and predictive biomarkers are also used in randomized clinical trials as stratification factors (to ensure balance between groups) and for covariate adjustment purposes (to increase power). Finally, adjustment for such biomarkers is important to render non-randomized groups more comparable (e.g. through propensity score methods, see Austin (16) and Brookhart et al. (17), or regression adjustment).

Third, on-study pharmacodynamic biomarkers capture the effect of the drug after its administration. The goal of these biomarkers is to demonstrate that the drug has the anticipated biological effect (i.e. establishes a proof of mechanism (PoM)). These biomarkers are usually linked to the MoA (e.g. the activation of natural killer cells, CD8 T cells during the treatment with Il15 (18)), but can be independent of it. A goal of analyses involving pharmacodynamic biomarkers is to relate the measured biological effect indicated by the biomarker itself to the observed clinical efficacy. In the optimal case, these biomarkers help in understanding the dose-response relationship and may be used as surrogates for clinical efficacy endpoints (formal surrogacy needs to be established with extensive subject level-data and specific methods (see Burzykowski et al. (19)).

Fourth, safety biomarkers are on-study biomarkers intended to measure the likelihood, presence, or extent of toxicity (e.g. IL6 serum levels in the context of cytokine release syndrome (CRS), see Pabst et al. (20) and Section 3.2). Detection of or change in a biomarker can allow dose modification or treatment interruption before toxicity becomes severe. These markers may also serve to characterize the dose-safety relationship.

Along with these biomarkers, the efficacy benefits that patients derive from a drug is measured through classical clinical endpoints. In early phase trials and for classical cytotoxic drugs for solid tumors, the benefit is typically measured in terms of tumor shrinkage induced by the drug. The tumor shrinkage has been classified into a response criteria by Therasse et al. (21). Even though long term benefit, typically measured by the time to death (or overall survival, abbreviated as OS), is not always associated to responses (RECIST criteria defined by Therasse et al. (21)), the goal of drugs other than immunotherapies remains to shrink the tumors. For drugs with an indirect effect on the tumors, like immunotherapies, tumor shrinkage may no longer be representative of the MoA and of long term clinical benefit (22). Therefore, endpoints like progression-free survival (PFS), which can capture the absence or a slow regrowth, and especially OS are more relevant to represent the benefit of immunotherapies. Also tumor growth kinetic models, based on the total tumor size, can provide additional insights in the clinical benefit of immunotherapies (23). The analysis of biomarkers and the link to these endpoints introduce additional complexities (reviewed in Section 3.5).

To illustrate the structure of the manuscript, **Table 2** connects the biomarker objectives described above with the statistical models and consideration that we cover below.

**Table 2:** Structure of the manuscript: Clinical Questions are depicted on the left.

Biomarker objectives	Biomarker types	Statistical considerations and models	
MoA/PoM	Longitudinal biomarkers	Data modeling & transformation (Section 3.1)	PK-PD models (Section 3.7) PD biomarker models (Sections 3.5, low dimensional/3.6, high dimensional)
Dose optimization			Models for dose finding (Section 3.2)
Characterization of adverse events			PD biomarker models (Sections 3.5/3.6)
Patient enrichment	Baseline biomarkers		Prognostic (Section 3.3) and predictive (Section 3.4) biomarkers

Type of biomarker and statistical considerations are given with their respective Sections. MoA stands for mechanism of action, PoM for proof of mechanism, PK for pharmaco-kinetic, PD for pharmaco-dymanic.

## 3 Statistical methodologies

This section provides an overview of relevant statistical considerations and models. Many of the described methods and statistical principles have not been developed specifically for CIT biomarkers but we believe that adherence to good statistical practice is particularly important in this context because of the complexity of questions related to the immune system and the variety of new biomarkers analyzed. We would also like to stress that prior to any statistical analysis, a statistical analysis plan should be written which describes the precise scientific questions and all planned analyses. While exploratory analysis also play an important role, they should still be guided by a predefined analysis strategy to avoid data dredging.

### 3.1 Statistical modeling of biomarker data

An important step prior to any statistical analysis is to have a thorough understanding of how the biomarker data were obtained. Additional data normalization might be required depending on the steps already performed during the data acquisition process. The awareness of these details ultimately guides the various statistical modeling considerations, e.g. the choice of distribution, the need for covariate adjustment, or the treatment of missing values. Let’s illustrate this with an example. In the field of immuno-oncology a technology called flow cytometry plays a key role in measuring immune cell markers. An important step in the data acquisition is called “manual gating” which is typically employed to classify single cells into discrete cell types based on emitted fluorescent signals. As a consequence, the readout for flow cytometry data are proportions, i.e. number of cells relative to some reference cell population. The statistical challenge is then to choose a model with adequate distributional assumptions. For example linear regression relating the dose of a drug with the change in the proportion of activated T cells (adjusted by baseline) assumes that the residual errors are normally, independently and identically distributed with mean zero. Oftentimes biomarker data do not conform with these standard statistical assumptions (e.g. flow cytometry data). Statistics offers three possible solutions to deal with this challenge: the first approach is to transform the original values using a deterministic function such that the standard statistical assumptions are approximately met. The logarithmic and square root functions are popular examples of data transformations. The Box-Cox transformation family (24) is often used to approach data transformation more systematically using statistical estimation techniques. A transformation particularly useful in the example outlined above is the empirical logit transformation which maps proportions monotonically to the whole real line while keeping the interpretation of regression coefficients simple (25). The second approach extends linear regression by allowing the linear model to be related to the response variable via a link function and by allowing the magnitude of the variance of each measurement to be a function of its predicted value (26). In the case of the flow cytometry example, a logit-link function and binomial mean-variance relationship is well suited; overdispersion is dealt with either by including a random effect to account for the between sample variation or by adding a dispersion parameter to the mean-variance relationship (the latter approach is referred to as the quasi-binomial approach). The third approach consists in avoiding any distributional assumption and resort to non-parametric methods. In our view, in the context of exploratory biomarker analysis, the data transformation approach has two key advantages: firstly, statistical inference based on standard linear regression is robust and goes hand-in-hand with familiar visualizations (e.g. boxplot and scatter plot), which in-turn facilitates communication of results to non-statisticians; secondly, transformed data can be readily used as input to a variety of advanced statistical methods and data mining tools.

Two additional complications of biomarker analyses are: First, measurements below the assay’s detection limit may occur. Such measurements should not be excluded from the analysis. If their frequency is low, *ad-hoc* approaches such as imputing them with, say, half of the detection limit may be sufficient but if they are frequent, more sophisticated methods such as treating them as left-censored may be preferable (27). Second, the assay may be impacted by measurement errors. In this case, models, such as those described in the next sections, need to account for the additional variability potentially bias induced by the measurement error. To elicit the components (e.g. distribution assumptions) needed for a proper handling of measurement error, one needs to have data, either within the dataset considered in the analysis (internal), or outside (external, e.g. a assay development dataset or preclinical experiments) in which the true value can be linked to the assay. Several models can then be implemented (see Carroll et al. (28) for a general reference).

### 3.2 Dose finding

The introduction of new mechanisms of action has led to new safety risks and challenges in the determination of the optimal dose to be administered. In this context, biomarkers are used to provide a more granular observation of the drug’s anti-tumor activity or safety and allow the determination of the dose based on a more precise benefit risk assessment (compared to clinical endpoints). In this section, we highlight some challenges of dose finding for immunotherapies and provide some options to optimize dose finding using biomarkers.

A typical issue with some classes of immunotherapies (T-cell engagers) is the cytokine release syndrome (CRS), a trigger of a systemic inflammatory response characterized by a large amount of cytokines being released. CRS limits the exploration of high and effective doses. A mitigation strategy was found by which one or more low priming doses (also called step-up) attenuate the cytokine release upon repeated doses and allow the subsequent administration of a high and effective target dose (Bartlett et al. (29)). The dose finding exercise then requires the determination of the priming doses and the target dose, as opposed to, for classical drugs, the determination of the tolerable target dose. Dose escalation designs have been proposed in this context by Xu et al. (30); Gerard et al. (31) and more recently by Dejardin et al. (32).

Other dose finding challenges are summarized by Wages et al. (33). Challenges include late onset toxicities, i.e. toxicities occurring beyond the typical observation period (set to 21-28 days after dosing). In the presence of late toxicities, one could prolong the observation period for each patient. However, this causes the dose escalation trial to be significantly prolonged in some cases. Wages et al. (33) reports dose escalation designs to allow partial observation of patients in the dose escalation decisions. Another challenge is the fact that higher doses may not be the best in terms of efficacy. Indeed, some drugs may have an optimal biological activity at intermediate dose ranges (“bell-shaped-response”) with suboptimal activity at higher doses, for which however toxicity may be observed. Dose escalation designs must therefore be constructed such that both safety and efficacy are optimized. Wages et al. (33) reports options to use both safety and efficacy in the determination of the next dose in a dose escalation design. These approaches work conditionally on the fact that the efficacy endpoint is available at the time of the dose escalation decision.

When optimizing on efficacy, a key question is the identification of the most appropriate function that describes dose-response relationship. A structured procedure to study the relationship with dose has been proposed by Bretz et al. (34); Nie et al. (35). In this procedure, a set of coefficients from candidate models are calculated, allowing different dose response shapes. Then, a multiple test procedure is implemented to select the dose response signals, followed by a model selection step. This procedure allows flexible modeling of the dose response signal, preserving the robustness to misspecification that are associated with multiple testing. A simpler approach consists in using a general model that can accommodate many different dose-response shapes. The EMACS model (see Seber and Wild (36) Section 7) is an example of such models.

### 3.3 Prognostic baseline biomarkers and models for clinical endpoints

The prognostic association of one or multiple baseline biomarkers with clinical outcomes is typically assessed using regression models, such as the logistic model for binary outcomes (e.g. the overall response rate) or the Cox proportional hazards model for time-to-event outcomes (e.g. PFS or OS). As described in Section 3.1 it is important that adequate transformations are applied to biomarkers before including them as covariates. If there’s uncertainty regarding the shape of the association between a biomarker and the outcome, spline models can easily be incorporated into regression models (Harrell (37)). In order to reduce the complexity of a multivariable regression model, naive step-wise variable selection methods are frequently used but have a number of issues including that they yield over-optimistic regression coefficients and *p*-values. More modern selection procedures such as the lasso, boosting, or Bayesian model averaging are recommended (Harrell (37)). To aid clinical decision making, medical practitioners are used to discretizing continuous biomarker into different risk categories by applying cut-points. A number of statistical pitfalls are associated with this practice. First, discretization introduces a substantial loss of information. Second, data-driven cut-point selection algorithms may lead to overfitting and an exaggerated association between the dichotomized biomarker and the clinical outcome. For a review of these pitfalls and guidance on the selection of cutpoints, we refer to Polley and Dignam (38) and (39, Chapter 16).

A dataset of sufficient size and quality is essential in order to develop a robust prognostic model (Riley et al. (40)). Sample sizes of early development studies are typically insufficient for this purpose and data from large cohort studies or confirmatory trials are required. Moreover, for most cancer types, a plethora of published prognostic models exist already. As an example, a systematic review of prognostic models in breast cancer identified 58 published models but only a few of them had been validated widely in different settings and, frequently, their performance was suboptimal in independent populations (Phung et al. (41)). For this reason, it may be scientifically more relevant to externally validate or update existing prognostic models in new contexts or to assess whether the addition of a new biomarker improves an already existing prognostic model (Steyerberg (39)).

We refer to Harrell (37); Steyerberg (39), and Collins et al. (42) for comprehensive resources to developing and reporting prognostic models and only highlight a few points relevant to performance assessment and validation below. First, it is important to characterize the performance and clinical utility of a prognostic model. Performance measures should report the overall performance of a model, its discrimination, i.e. its ability to discriminate subjects with an event from those without an event, as well as its calibration, i.e. the agreement between observed outcomes and predictions. Frequently reported measures for these three performance aspects are the Brier score, the concordance probability or *c*-index, and calibration plots. For a detailed review of performance measures, we refer to (39, Chapter 15).

A prognostic model is internally valid (or “reproducible”) if its reported performance adequately reflects the actual performance of the model that would be observed in an independent random data sample from the same source population as the dataset used for model development. One reason why a prognostic model may not be reproducible is that performance is overestimated due to overfitting. A common reason for this is that model development and performance assessment are performed on the same dataset. If one builds a complex prognostic model and then evaluates its performance in the same dataset, then one may obtain a very good accuracy on that dataset, but that is not a fair assessment of the future performance of the model. Rather, the appropriate question is whether this prognostic model will provide a sufficient level of accuracy to be of use when applied to a truly independent test set. In internal validation studies where only one dataset is used for model development and validation, there are two main options to mimic performance in an independent dataset. The first option is to reserve a fixed portion of the dataset (e.g. 20%) for validation. That is, model development is based on training data, i.e. the data not included in the validation dataset, and a hold-out validation dataset is strictly shielded from access during model development. Once model development is completed, its performance is evaluated in the independent hold-out dataset. Since this approach discards a part of the dataset for training, it has some drawbacks (see Section 17.2.2.2 in Steyerberg (39)), one of which is the stability of the findings. Therefore, a second option is to implement internal validation using re-sampling approaches such as cross-validation or bootstrapping. These approaches include repeatedly splitting the data into a training and a validation data set, developing the model including parameter estimation in the training data set, and then assessing performance in the validation dataset. In general, cross-validation and bootstrapping are more efficient approaches to assess average model performance than a single random data split (see Steyerberg (43) and the discussion about prediction stability and error in boostrap sample in Riley and Collins (44).

The above steps outline the discovery and development stages of prognostic markers which includes the identification of prognostic biomarkers or the building of a prognostic model, and their performance assessment in internal validation studies. The full development of such biomarkers or models for clinical use is typically split into three phases (Moons et al. (45); Ou et al. (46)): discovery or development studies, external validation studies, and clinical impact studies. Analytical validation of the biomarker is another important phase that is outside of the scope for this article (Ou et al. (46); Kraus (47)). The purpose of external validation studies is to confirm the performance of the biomarker/prognostic model in a completely independent dataset and to assess its generalizability to a wider population. Finally, impact studies assess whether the use of the biomarkers/prognostic model by practicing doctors actually improves clinical outcomes. Ideally, impact studies are randomized controlled trials which compare biomarker-guided treatment with the standard of care.

### 3.4 Predictive baseline biomarkers for clinical endpoints

The exploratory assessment whether a baseline biomarker is predictive, i.e. whether it impacts the magnitude or direction of a treatment effect, typically requires data from randomized controlled clinical trials. Predictive biomarker effects can be modeled by including a *treatment* × *biomarker* interaction term in the regression model. Of note, logistic and Cox regression model the *relative* reductions in the odds or rate of an event. If a biomarker is prognostic, then the *absolute* risk reduction may vary across biomarker levels even if the relative risk reduction is the same. Standard analyses to assess whether a biomarker is predictive are statistical tests for the *treatment* × *biomarker* interaction term as well as forest plots which visualize treatment effects in subgroups defined by the biomarker (Alosh et al. (48)). For a tutorial on data-driven identification of predictive subgroups we refer to Lipkovich et al. (49).

Limitations of exploring several biomarkers and clinical variables for predictive effects are well-recognized: an increased risk of false-positive findings due to multiplicity combined with an increased risk of false-negative findings and highly variable treatment effect estimates in subgroups due to the limited sample size and power in subgroups. Therefore, findings from such analyses should only be considered credible if additional criteria are fulfilled, e.g. that the predictive effect of the biomarker was correctly pre-specified (including the direction of the effect), that the predictive effect is supported by prior evidence, that only a low number of potential predictive markers was explored (ideally 3 or fewer), that chance cannot explain the finding, and that arbitrary cut points were avoided (Schandelmaier et al. (50)). Otherwise, the findings should be considered exploratory and hypothesis-generating only.

For the confirmatory assessment of a predictive biomarker and its inclusion in a drug label (e.g. the restriction of a label to a biomarker-positive subgroup or a claim of enhanced efficacy in this subgroup), a randomized clinical trial with type I error control across the all-comers population and the biomarker-positive subgroups is typically required. As an example, the IMpassion130 trial, a randomized phase 3 trial of the CPI atezolizumab in advanced triple-negative breast cancer, hierarchically tested the clinical endpoints first in the all-comers (intention-to-treat) population and then in the PD-L1–positive subgroup (Schmid et al. (51)). As another example, the IMpassion031 trial of the same molecule in early-stage triple-negative breast cancer was an adaptive enrichment trial with two primary populations (PD-L1 positives and all-comers) allowing for population section at an interim analysis (Nguyen Duc et al. (52); Mittendorf et al. (53)).

### 3.5 Models for associations between on-treatment biomarkers and clinical endpoints

In this section, we discuss models which analyze the impact of one or a low number of biomarkers measured on-treatment, i.e. pharmacodynamic biomarkers, on clinical outcome. A key aspect of on-study biomarkers is the timing at which measures are taken. For some biomarkers, repeated measurements are available (e.g. parameters taken in the blood) while tumor parameters (requiring invasive techniques or tumor imaging data, for which the amount of radiation received by the patient is a limiting factor) are usually taken only once after treatment start. The timing aspects need to be explicitly accounted for in the analyses, which, in our experience, is often not done.

Before discussing appropriate analysis methods for on-treatment biomarkers, we list five additional challenges which are important to consider while planning the analyses and writing a statistical analysis plan. First, it is critical to avoid immortal-time bias in the analysis (Mantel and Byar (54)). This occurs if a clinical endpoint that is timed from study enrolment is compared across groups defined by a classifying event occurring during follow-up e.g. by an on-treatment biomarker. A classical example of this bias in oncology is the naive comparison of overall survival by tumor response categories (Anderson et al. (55)). More generally, any analysis which treats an on-treatment biomarker as if it was a baseline biomarker or which reverses time, i.e. tries to explain the past with the future, should be avoided.

Second, when studying the relationship between the risk of a clinical event (response or progression) and the longitudinal evolution of the biomarker, models need to capture the impact of changes in the biomarker on the risk of the event. This implies that the biomarker and the clinical event need to be modeled jointly across time. An example of this type of analysis is the construction of a dynamic prognostic score for the risk of recurrence of prostate cancer depending on longitudinal assessments of a prostate-specific antigen (PSA) biomarker (56).

Third, considerations regarding which aspects of the longitudinal biomarker dynamics affect the clinical endpoint are important. In the previous example, the current value of the PSA marker may directly affect the rate of the clinical event. In other settings, there might be a lag-effect, i.e. the risk of event at time *t* is influenced by the biomarker at time *t* − *ℓ* for a fixed or variable lag time *ℓ*. Alternatively, the impact of the rate of change or the accumulation of a biomarker is of primary interest. In the latter setting, the biomarker’s area under the (time-)curve (AUC) may be an alternative as the covariate of primary interest.

Fourth, competing events such as adverse events requiring treatment interruptions, may occur which affect either the interpretation or the existence of subsequent measurements of the biomarker and the clinical event. It is typically not plausible that the occurrence of such adverse events is independent of on-treatment biomarkers and clinical endpoint. For example, the biological activity of the drug may induce a change in the biomarker and in the clinical endpoint, but at the same time lead to toxicity. In such cases, the joint evaluation of the biomarker and the clinical endpoint is impacted by the competing risk of an interruption of treatment due to the toxicity which needs to be accounted for in the analysis.

Fifth, the biological variation of a biomarker may vary substantially by subject or by dose. Typically the protocol defined sampling schedule is common to all patients and dose levels. As a consequence, the biomarker measurements may not be taken at the same time relative to the biological process unfolding in each patient. An example of a biomarker where this issue arises are natural killer (NK) cells measured in blood: these immune cells go through a marginalization process at which time they become non observable in the blood (see Conlon et al. (18)).

These considerations may be addressed using the analysis methods presented below.

For biomarkers measured infrequently (e.g. via biopsies), landmark analyses may be used (Van Houwelingen (57)). In such analyses, a landmark time is defined as the time of a specific biomarker measurement which defines a new baseline (time zero) for subsequent analysis. That is, the value of the biomarker at the landmark is treated as a baseline covariate, subsequent biomarker measurements are ignored, the clinical time-to-event endpoint is re-defined based on the new time origin, and all subjects with a clinical event or censoring prior to the landmark are excluded from the analysis. Subsequently, standard regression methods for modeling the clinical endpoints such as Cox regression are used. Landmark analysis avoid immortal-time bias but care needs to be taken when comparing randomized treatment groups because they are no longer comparable after conditioning on the landmark.

In order to model longitudinal biomarkers and clinical endpoints simultaneously, joint models are popular (Rizopoulos (58)). These models have two components, a longitudinal model for the biomarker (typically a linear mixed effects model) and a time-to-event model for the clinical outcome (e.g. a Cox model), and shared parameters which determine how the biomarker evolution affects the rate of clinical event. Such models allow that the actual (measurement-error free) value of the biomarker, or a lagged value, or a rate of change, or the area under the curve is included as a covariate for modeling the clinical event. Alternatively, when relevant thresholds are defined for the biomarker values, the subject’s evolution through different states of biomarker categories and clinical outcomes can be modeled using multi-state models (59). Multi-state models are also useful to address the competing risk problem described above.

In the context of randomized trials, relevant clinical questions may arise regarding the treatment effect in subpopulations of patients, which could experience certain clinical or disease related events post-randomization. As an example, subjects in the CIT group may experience anti-drug antibodies and it is of interest to know whether this affects CIT efficacy. A principal stratification strategy can be applied to these events to define a causal treatment effect (Bornkamp et al. (60)). Specifically, Kong et al. (61) describe an approach where subjects with an ADA in the CIT arm are compared to the control arm which is re-weighted based on relevant baseline characteristics to be rendered more comparable to the ADA-positive CIT subpopulation. These weighting approaches for principal stratification are similar to inverse probability of treatment weighting (IPTW) approaches which are frequently used to address causal questions, e.g. to determine the treatment effect in subgroups defined by baseline biomarkers in the presence of additional confounding variables [see Austin and Stuart (62)].

To address the confounding effect linked to biological variability of the biomarker, an option is to leverage the (semi) mechanistic models described in Section 3.7. These models provide a more complete description of the longitudinal profile, that may be patient- and covariate- dependent (e.g. to dose) compared to measurements at a fixed time for all patients. Using these profile, quantities such as the maximum value over a period of time, or the AUC are more robustly estimated. A two-stage approach combining the mechanistic model and models such as the landmark or the joint models can be used to study in a more robust manner the relationship between the clinical endpoint and the biomarker.

In addition to these specific models, one may need to take potential imbalances in value of the biomarker at baseline into account. These imbalances may lead to false conclusions on the impact of the treatment on the on-study biomarker value. Authors [see Vickers (63), (64, Section 2.4)] suggest to incorporate the baseline value in models of the on-study value. This conclusion is also true when baseline values are partially missing [see Kenward et al. (65)].

### 3.6 Hypothesis generation and high-dimensional statistics

Besides testing key biomarkers hypothesis (as usually specified in the study protocol) early clinical development many times involves extensive exploratory data analysis work. The aim of these analysis efforts is to generate new hypotheses related to MoA, PoC (proof of concept) and patient selection/enrichment. Omics-based technologies typically measure thousands of molecular features (e.g. genomics, transcriptomics and proteomics) in parallel. An important characteristics of such high-dimensional data is that the number of features *p* is typically much larger compared to the number of samples *n* (e.g. number of patients). Such data sets present a variety of statistical challenges, since classical theory and methodology can break down in surprising and unexpected ways Buhlmann¨ and Van De Geer (66), Wainwright (67), Hastie et al. (68). Hypothesis generation based on high-dimensional biomarker data can be typically divided into three types of analysis tasks:

The first task considers *p* biomarkers and one response variable and the aim is prediction and variable selection. Such analysis can be the starting point of the development of a novel prognostic or predictive biomarker which then could potentially be used for patient selection/enrichment (see section 3.3 on the prognostic/predictive validation of biomarker). Numerous computational approaches have been developed for this purpose which adequately address overfitting and simultaneously provide information on variable importance. These methods use modern statistical concept such as such as L1/L2-regularization (e.g. Ridge-, Lasso Regression), bootstrap aggregation (Random forest) and boosting. A recent example is the construction of a polygenic risk scores (PRS) for anti-PD-L1 induced hypothyroidism and the subsequent assessment of PRS variant importance using Lasso Regression [Khan et al. (69)].

The second task considers one (or few) potential explanatory variable whereas the *p* biomarkers take the role of response variables. The aim is to identify those biomarkers which show a relationship with the explanatory variable. An example is the exploration of dose relationship with molecular PD markers (e.g. as measured by flow cytometry or gene expression). This involves simultaneous testing of many hypothesis. It is essential to perform multiple testing correction in order to correct for occurrence of false positives [Dudoit et al. (70)]. In addition, empirical Bayes approaches which borrow information across the *p* biomarkers have been shown to be superior in small *n* setting compared to a one-biomarker-at-a-time analysis [Smyth (71)].

The third task explores commonalities across the samples at the level of the *p* biomarkers. For example exploring the molecular heterogeneity of patients can provide new insights on the disease biology and the mode of action of a new molecular entity. Several approaches have been developed for unsupervised learning in the *p* >> *n* setting [Monti et al. (72), Städler et al. (73)]. In addition, dimensionality reduction methods such as principle component analysis (PCA), multidimensional scaling (MDS) and t-distributed stochastic neighbor embedding (t-SNE) are frequently used to visualize the high-dimensional data with fewer dimensions and identify patters. In a recent example such statistical approaches helped to decipher the single-cell level phenotypical and transcriptional consequences of treatment with anti-PD-1 and with PD1-TIM3 and PD1-LAG3 bispecific antibodies [Natoli et al. (74)].

### 3.7 PK/PD modeling

Longitudinal mathematical models are powerful tools that describe temporal changes of a biomarker upon treatment administration and therefore leverage the entirety of the data collected during a study, without the need to rely on a single time-point (landmark analysis) or to discretize data. Here we focus on top-down population pharmacokinetic (PK)/PD models which are predominantly built on observed clinical data and are mostly empirical or semi-mechanistic. These non-linear mixed effect models describe the relationship between the pharmacokinetics of the drug (e.g. the plasma concentration-time course) and the dynamics of a response variable. The model structure is formulated via a set of differential equations defined by estimated model parameters. The model includes statistical distributions (e.g. normal, log-normal) representing between-patient variability in both PK and PD parameters, which can be quantified and distinguished from residual unexplained variability. The PK component describes how the drug is absorbed, distributed, and cleared from the body, including target-mediated drug disposition when applicable. The PD component offer a simplified mathematical representation of the patho-physiological processes relevant to biomarker response. A link function between PK and PD characterizes the exposure-response relationship and quantifies the part of the biomarker response variability that can be explained by variability in PK. In addition, covariates such as patient baseline characteristics can be evaluated during model building to explain (part of) the variability in PK and PD. By further including additional components associated to clinical response or safety outcomes of interest, modeling frameworks can be built to answer specific questions (fit-for-purpose). Model-based simulations can then be used for *in-silico* exploration of untested dosing scenarios that can be further evaluated in the clinic, project long-term response, or make prospective predictions for a new patient population. Published examples (Ribba et al. (75), Silber Baumann et al. (76), Netterberg et al. (77), Chen et al. (78)) show the potential of PK/PD models to help the selection of doses and schedules (e.g. induction-maintenance doses, priming doses) to maximize efficacy and/or mitigate safety risks of CIT. These models may be updated during clinical drug development based on new arising data and questions (learn and confirm paradigm). Of note, identification of relevant biomarkers based on exploratory data analysis are a prerequisite to the development of such models. In addition, informative schedule of assessments for PK and biomarker, and timely availability of data to modeling teams, are key elements to the ensure a meaningful delivery of modeling outputs for decision making. Other mathematical modeling approaches, including mechanistic bottom-up and middle-out approaches that rely less on clinical data are out of scope but have been recently reviewed elsewhere (79).

## 4 Discussion

Well conducted biomarker-driven clinical trials can increase the success rates in drug development. However, a critical condition for success is that relevant prognostic or predictive baseline biomarkers or pharmacodynamic on-treatment biomarkers for clinical efficacy or safety outcomes can be identified. Despite a plethora of examples and some success stories (see **Table 1** in **Supplemental Material**), the identification of an optimal biomarker proves extremely challenging, most likely due to the complex and dynamic interplay between the tumor and the host immune system. Many publications identify new biomarkers but there are much fewer studies which aim to demonstrate their clinical validity and utility and fewer again enter clinical practice. Moreover, a number of research studies have highlighted deficiencies in some areas of biomarker research. As an example, Malats et al. (10) concluded in a systematic review of 168 publications from 117 studies that despite all of this research, there is still no sufficient evidence to conclude whether changes in p53 act as markers of outcome in patients with bladder cancer. More recently, a systematic review of Kempf et al. (11) found evidence of frequent overinterpretation of findings of prognostic factor assessment in high-impact medical oncology journals.

In the introduction, we introduced four key challenges in the development of CIT: 1) demonstration of MoA, 2) dose selection and optimization, 3) mitigation and prevention of immune related adverse events and 4) patient enrichment and patient selection. A major complexity of CIT development is the indirect targeting of the tumor through the immune system which requires a better understanding of the immune system and how it is impacted by the drug. Biomarker technologies provide tools that can support addressing these key challenges. In this manuscript, we reviewed important methodological aspects in the analysis of biomarkers, (summarized in **Supplementary Table 2**). We covered basic aspects of biomarker modeling, analyses of low-dimensional baseline and on-treatment biomarkers, high-dimensional analysis, and topics related to dose finding and PK/PD modeling.

This article covers a broad range of statistical methods for biomarker analyses relevant to the early development of cancer immunotherapies. However, we also acknowledge some limitations. First, we did not provide a detailed description of statistical methods and their relative merits, as this would have required extending the article substantially. Instead, we aimed to provide an overview of methods, highlight potential pitfalls, and give relevant references that cover these methods in-depth. Second, analytical validation of biomarkers is not covered in this article at all (Ou et al. (46), Kraus (47)). Third, our article primarily focuses on an early development program setting where biomarkers are analyzed for exploratory and hypothesis-generating purposes. In such settings, study designs (e.g., dose escalation designs covered in Section 3.2) are usually not specifically designed for biomarker analyses. We have only briefly discussed the important topic of confirmatory randomized clinical trials which are required to establish the clinical utility of baseline biomarkers at the end of Sections 3.3 and 3.4. Important design options at this stage include biomarker-stratified designs, enrichment designs, and biomarker-strategy designs [Freidlin et al. (80), Tajik et al. (81)]. A general overview of the strategic use of biomarkers as a drug development tool and regulatory pathways is provided by Kraus (47).

The aim of this manuscript was to highlight key statistical consideration and present valid analysis methods for the identification and evaluation of biomarkers relevant to early clinical development in the CIT field. We hope that it supports data scientists and other drug developers to derive robust and reproducible conclusions.
