# Supplementary material for: An overview of statistical methods for biomarkers relevant to early clinical development of cancer immunotherapies
Source: Front Immunol. 2024 Aug 21;15:1351584. doi: 10.3389/fimmu.2024.1351584 (PMC11371698; doi:10.3389/fimmu.2024.1351584)
Supplement: Supplementary file 1 [file DataSheet1.pdf]

Supplemental material

An overview of statistical methods for biomarkers relevant to early clinical development of cancer immunotherapies

David Dejardin, Anton Kraxner, Emilie Schindler, Nicolas Städler, Marcel Wolbers.

Examples of biomarkers for cancer immunotherapies

Table 1: Biomarker examples

Biomarker	Measurement method	Exemplary findings & considerations
PD-L1	IHC	• May be driven by IFN gamma following T-cell infiltration (Shuming Chen et al., 2019)<br>• Imperfect, but commonly used biomarker for selecting patients for CPI therapy across tumor types (Doroshow et al., 2021; Vaddepally et al., 2020)<br>• Different companion diagnostic assays with different PD-L1 antibodies, thresholds of positivity, and scoring systems as challenge (Tsao et al., 2018)
TMB	DNA sequencing (WES, targeted gene panels)	• May indicate presence of immunogenic mutation-associated neoantigens (Fumet et al., 2020; Wei et al., 2018)<br>• Improved response with anti-PD1/PD-L1 therapies (Goodman et al., 2017; Rizvi et al., 2015; Snyder et al., 2014)<br>• Lack of a standardized approach to determine TMB
dMMR/MSI	IHC, qPCR, NGS	• May indicate presence of immunogenic mutation-associated neoantigens (Germano et al., 2017)<br>• Efficacy of CPI has been demonstrated in MSI-H/dMMR colorectal cancers, endometrial cancers and gastric cancers leading to approval of Pembrolizumab in MSI-H/dMMR tumors (Le et al., 2015; Overman et al., 2017)<br>• Lack of standardization in microsatellite panels and thresholds

Continued on next page

Biomarker	Measurement method	Exemplary findings & considerations
Gene expression profiling<br>(e.g. T effector, IFN-g based signatures)	RNA sequencing	• Can predict response to CPI (Ayers et al., 2017; Danaher et al., 2018; Fehrenbacher et al., 2016; Jamieson et al., 2017)<br>• Can be easily applied to existing datasets<br>• Lack of resolution & spatial context (may be overcome by emerging single cell technologies, deconvolution algorithms or spatial transcriptomic approaches)
Tumor infiltrating lymphocytes (TIL)<br>(e.g. CD8 density, PD-1, Granzyme B, tertiary lymphoid structures)	IHC, IF	• Immunophenotyping of tumors may provide crucial novel prognostic information and is associated with improved outcome (Cabrita et al., 2020; Galon et al., 2012; Zeng et al., 2016)<br>• mIHC/IF assays outperformed other biomarkers including PD-L1, IHC, TMB, and GEP (Lu et al., 2019)<br>• Assessment of spatial relationships and protein coexpression on specific cellular subsets as main strength (multimarker approaches)
Peripheral T cell subsets<br>(e.g. proliferating, Teff/mem, PD-1+)	Flow cytometry, CyTOF	• The first-week proliferative response of peripheral blood PD-1+CD8+ T cells predicts the response to anti-PD-1 therapy in solid tumors (Kim et al., 2019)<br>• Functional systemic CD4 immunity is required for clinical responses to PD-L1/PD-1 blockade therapy. (Zuazo et al., 2019)<br>• Multi-marker approaches key to precisely assess specific cellular subsets & co-expression patterns<br>• Limited sample stability & low event counts for rare subpopulations as main challenges along others

Continued on next page

Biomarker	Measurement method	Exemplary findings & considerations
Plasma cytokines (e.g. IFN-g, IL-6)	Immuno-assay	• Increased levels of IFN-γ is associated with improved outcome to CPI (M. Wang et al., 2021)<br>• Anti-cytokine drug, tocilizumab has been a cornerstone in the treatment of CAR-T-associated CRS through its ability to dampen CRS (Genentech, 2017)<br>• Unlikely a single cytokine will be sufficient to predict immunotherapy response and irAEs for the complexity of TME and interaction between cytokines
Blood cell populations (lymphocyte, neutrophil counts)	Hematology	• A baseline signature of a low absolute neutrophil count, high absolute lymphocyte counts and high absolute eosinophil count was associated with a better outcome of nivolumab treatment (Tanizaki et al., 2018)<br>• Clinical routine assessment broadly available
Inflammatory mediators (e.g. CRP)	Blood chemistry, Immuno-assays	• C-reactive protein reduction post treatment is associated with improved survival in atezolizumab treated NSCLC patients (Nardone et al., 2021; Patil et al., 2021)<br>• Clinical routine assessment broadly available
T cell repertoire	TCR sequencing	• TCR repertoire diversity of peripheral PD-1+CD8+ T cells predicts clinical outcomes after immunotherapy in patients with (Han et al., 2020)
Tumor markers (e.g. CA125, PSA)	Immuno-assay	• PSA response as potential surrogate efficacy endpoint in prostate cancer treated e.g. with CPI (Powers et al., 2020)

Continued on next page

Biomarker	Measurement method	Exemplary findings & considerations
ctDNA	DNA sequencing (WES, targeted gene panels)	• Early changes in plasma ctDNA levels can predict response to CPI in NSCLC (Vega et al., 2022)<br>• Need to prove clinical utility, decreasing turnaround time, decreasing costs, and improving sensitivity and specificity for MRD (Dang et al., 2022)
Gene mutations (e.g. EGFR, BRCA)	DNA sequencing (WES, targeted gene panels)	• Activating EGFR mutations have been associated with dramatic responses to first-generation EGFR-TKIs<br>• NSCLC patients with wild-type EGFR or a KRAS mutation derived a significant OS benefit from nivolumab (Borghaei et al., 2015)
Others	Multiple approaches	• Imaging (e.g. PET, CT)<br>• Gene polymorphisms (e.g. HLA, KIR)<br>• Circulating tumor cells<br>• Metabolites<br>• Microbiome

List of statistical methods discussed in the manuscript

Table 2: List of statistical methods discussed in the manuscript

Method	Reference
Section 3.1 Statistical modelling of biomarker data	
Box-Cox transformation	Box et al., 1964
Logit transformation	Warton et al., 2011
Generalized linear models	McCullagh et al., 2019
Detection limits	Helsel, 2005
Measurement error	R. J. Carroll et al., 1995
Section 3.2 Dose Finding	
Dose escalation design for cytokine release syndrome	Dejardin et al., 2024; Gerard et al., 2021; Xu et al., 2021
Dose escalation with late toxicities	Wages et al., 2018
Dose escalation with efficacy optimization	Wages et al., 2018
MCPMOD	Bretz et al., 2005; Nie et al., 2016
EMACS model	Seber et al., 2003
Section 3.3 Prognostic baseline markers and models	
Model development, validation, and reporting	Collins et al., 2015; Harrell, 2015; E. Steyerberg, 2019
Sample size calculation	Riley et al., 2020
Lasso, boosting, Bayesian model averaging for model development	Harrell, 2015
Spline models	Harrell, 2015
Cut-point selection	Polley et al., 2021; E. Steyerberg, 2019, chapter 16
Performance measures (Brier score, c-index, calibration plots)	E. Steyerberg, 2019, chapter 15
Cross-validation	E. W. Steyerberg, 2018
Phases of biomarker/prognostic model development	Moons et al., 2009; Ou et al., 2021
Section 3.4 Predictive baseline biomarker	
Subgroup analysis in clinical trials	Alosh et al., 2017
Data-driven subgroup analysis	Lipkovich et al., 2017
Credibility criteria for subgroups	Schandelmaier et al., 2020
Adaptive enrichment trials	Nguyen Duc et al., 2021
Section 3.4 Models for association between on-treatment biomarkers and clinical endpoints	

Continued on next page

Immortal-time bias	Anderson et al., 1983; Mantel et al., 1974
Landmark analysis	Van Houwelingen, 2007
Joint models	Rizopoulos, 2012
Multi-state models	Putter et al., 2007
Principal stratification, comparison between subpopulations based on on-treatment biomarkers	Bornkamp et al., 2021; Kong et al., 2022
Section 3.5 Hypothesis generation and high-dimensional statistics	
Model Selection	Khan et al., 2021
Multiple testing correction	Dudoit et al., 2008
Borrowing across biomarkers	Smyth, 2004
Unsupervised learning	Monti et al., 2003; Städler et al., 2017
3.6 PK/PD modeling	
Models for impact of dose on immune cell subsets	X. Chen et al., 2019; Netterberg et al., 2019; Ribba et al., 2018; Silber Baumann et al., 2018
Mechanistic approaches	Valentinuzzi et al., 2020

References

Alosh, Mohamed, Mohammad F Huque, Frank Bretz, and Ralph B D'Agostino Sr (2017). "Tutorial on statistical considerations on subgroup analysis in confirmatory clinical trials". In: *Statistics in medicine* 36.8, pp. 1334–1360.

Anderson, James R, Kevin C Cain, Richard D Gelber, et al. (1983). "Analysis of survival by tumor response". In: *J Clin Oncol* 1.11, pp. 710–719.

Ayers, Mark, Jared Lunceford, Michael Nebozhyn, Erin Murphy, Andrey Loboda, David R Kaufman, Andrew Albright, Jonathan D Cheng, S Peter Kang, Veena Shankaran, et al. (2017). "IFN-γ–related mRNA profile predicts clinical response to PD-1 blockade". In: *The Journal of clinical investigation* 127.8, pp. 2930–2940. DOI: 10.1172/JCI91190.

Borghaei, Hossein, Luis Paz-Ares, Leora Horn, David R Spigel, Martin Steins, Neal E Ready, Laura Q Chow, Everett E Vokes, Enriqueta Felip, Esther Holgado, et al. (2015). "Nivolumab versus docetaxel in advanced nonsquamous non–small-cell lung cancer". In: *New England Journal of Medicine* 373.17, pp. 1627–1639. DOI: 10.1056/NEJMoa1507643.

Bornkamp, Björn, Kaspar Rufibach, Jianchang Lin, Yi Liu, Devan V Mehrotra, Satrajit Roychoudhury, Heinz Schmidli, Yue Shentu, and Marcel Wolbers (2021). "Principal stratum strategy: potential role in drug development". In: *Pharmaceutical Statistics* 20.4, pp. 737–751.

Box, George EP and David R Cox (1964). “An analysis of transformations”. In: *Journal of the Royal Statistical Society: Series B (Methodological)* 26.2, pp. 211–243.

Bretz, F., J. C. Pinheiro, and M. Branson (2005). “Combining Multiple Comparisons and Modeling Techniques in Dose-Response Studies”. In: *Biometrics* 61.3, pp. 738–748. DOI: https://doi.org/10.1111/j.1541-0420.2005.00344.x.

Cabrita, Rita, Martin Lauss, Adriana Sanna, Marco Donia, Mathilde Skaarup Larsen, Shamik Mitra, Iva Johansson, Bengt Phung, Katja Harbst, Johan Vallon-Christersson, et al. (2020). “Tertiary lymphoid structures improve immunotherapy and survival in melanoma”. In: *Nature* 577.7791, pp. 561–565. DOI: 10.1038/s41586-019-1914-8.

Carroll, Raymond J, David Ruppert, and Leonard A Stefanski (1995). *Measurement error in nonlinear models*. Vol. 105. measurment-error. CRC press.

Chen, Shuming, George A Crabill, Theresa S Pritchard, Tracee L McMiller, Ping Wei, Drew M Pardoll, Fan Pan, and Suzanne L Topalian (2019). “Mechanisms regulating PD-L1 expression on tumor and immune cells”. In: *Journal for immunotherapy of cancer* 7.1, pp. 1–12.

Chen, Xiaoying, Cris Kamperschroer, Gilbert Wong, and Dawei Xuan (2019). “A modeling framework to characterize cytokine release upon T-cell–engaging bispecific antibody treatment: methodology and opportunities”. In: *Clinical and translational science* 12.6, pp. 600–608.

Collins, Gary S, Johannes B Reitsma, Douglas G Altman, and Karel GM Moons (2015). “Transparent reporting of a multivariable prediction model for individual prognosis or diagnosis (TRIPOD): the TRIPOD statement”. In: *Annals of internal medicine* 162.1, pp. 55–63.

Danaher, Patrick, Sarah Warren, Rongze Lu, Josue Samayoa, Amy Sullivan, Irena Pekker, Brett Wallden, Francesco M Marincola, and Alessandra Cesano (2018). “Pan-cancer adaptive immune resistance as defined by the Tumor Inflammation Signature (TIS): results from The Cancer Genome Atlas (TCGA)”. In: *Journal for immunotherapy of cancer* 6.1, pp. 1–17. DOI: 10.1186/s40425-018-0367-1.

Dang, Donna K, Ben H Park, et al. (2022). “Circulating tumor DNA: Current challenges for clinical utility”. In: *The Journal of Clinical Investigation* 132.12. DOI: doi.org/10.1172/JCI154941.

Dejardin, David, Bo Huang, Ying Yuan, Ulrich Beyer, Jane Fridlyand, and Jiawen Zhu (2024). “Dose Optimization for Novel Oncology Agents: Design Options and Strategies”. In: *Statistics in Biopharmaceutical Research* 0, pp. 1–12. DOI: 10.1080/19466315.2024.2308856.

Doroshow, Deborah Blythe, Sheena Bhalla, Mary Beth Beasley, Lynette M Sholl, Keith M Kerr, Sacha Gnjatic, Ignacio I Wistuba, David L Rimm, Ming Sound Tsao, and Fred R Hirsch (2021). “PD-L1 as a biomarker of

response to immune-checkpoint inhibitors”. In: *Nature reviews Clinical oncology* 18.6, pp. 345–362. DOI: 10.1038/s41571-021-00473-5.

Dudoit, Sandrine, Mark J Van Der Laan, and Mark J van der Laan (2008). *Multiple testing procedures with applications to genomics*. Springer.

Fehrenbacher, Louis, Alexander Spira, Marcus Ballinger, Marcin Kowanetz, Johan Vansteenkiste, Julien Mazieres, Keunchil Park, David Smith, Angel Artal-Cortes, Conrad Lewanski, et al. (2016). “Atezolizumab versus docetaxel for patients with previously treated non-small-cell lung cancer (POPLAR): a multicentre, open-label, phase 2 randomised controlled trial”. In: *The Lancet* 387.10030, pp. 1837–1846. DOI: 10.1016/S0140-6736(16)00587-0.

Fumet, Jean-David, Caroline Truntzer, Mark Yarchoan, and Francois Ghiringhelli (2020). “Tumour mutational burden as a biomarker for immunotherapy: current data and emerging concepts”. In: *European Journal of Cancer* 131, pp. 40–50. DOI: 10.1016/j.ejca.2020.02.038.

Galon, Jérôme, Franck Pagès, Francesco M Marincola, Helen K Angell, Magdalena Thurin, Alessandro Lugli, Inti Zlobec, Anne Berger, Carlo Bifulco, Gerardo Botti, et al. (2012). “Cancer classification using the Immunoscore: a worldwide task force”. In: *Journal of translational medicine* 10, pp. 1–10. DOI: 10.1186/1479-5876-10-205.

Genentech, Inc (2017). *Tocilizumab US package insert*. URL: https://www.accessdata.fda.gov/drugsatfda_docs/label/2017/125276s114lbl.pdf.

Gerard, Emma, Sarah Zohar, Christelle Lorenzato, Moreno Ursino, and Marie-Karelle Riviere (2021). “Bayesian modeling of a bivariate toxicity outcome for early phase oncology trials evaluating dose regimens”. In: *Statistics in Medicine* 40.23, pp. 5096–5114. DOI: https://doi.org/10.1002/sim.9113.

Germano, Giovanni, Simona Lamba, Giuseppe Rospo, Ludovic Barault, Alessandro Magrı, Federica Maione, Mariangela Russo, Giovanni Crisafulli, Alice Bartolini, Giulia Lerda, et al. (2017). “Inactivation of DNA repair triggers neoantigen generation and impairs tumour growth”. In: *Nature* 552.7683, pp. 116–120. DOI: 10.1038/nature24673.

Goodman, Aaron M, Shumei Kato, Lyudmila Bazhenova, Sandip P Patel, Garrett M Frampton, Vincent Miller, Philip J Stephens, Gregory A Daniels, and Razelle Kurzrock (2017). “Tumor mutational burden as an independent predictor of response to immunotherapy in diverse cancerstmb predicts response to immunotherapy in diverse cancers”. In: *Molecular cancer therapeutics* 16.11, pp. 2598–2608. DOI: 10.1158/1535-7163.MCT-17-0386.

Han, Jiefei, Jianchun Duan, Hua Bai, Yuqi Wang, Rui Wan, Xin Wang, Si Chen, Yanhua Tian, Di Wang, Kailun Fei, et al. (2020). “TCR Repertoire Diversity of Peripheral PD-1+ CD8+ T Cells Predicts Clinical Outcomes after Immunotherapy in Patients with Non–Small Cell Lung CancerPe-

ripheral TCR Repertoire as a Biomarker for Immunotherapy". In: *Cancer immunology research* 8.1, pp. 146–154. DOI: 10.1158/2326-6066.CIR-19-0398.

Harrell, Frank E (2015). *Regression modeling strategies (second edition).* Springer Series in Statistics.

Helsel, Dennis R (2005). "More than obvious: better methods for interpreting nondetect data". In: *Environmental science & technology* 39.20, 419A–423A.

Jamieson, Nigel B and Ajay V Maker (2017). "Gene-expression profiling to predict responsiveness to immunotherapy". In: *Cancer gene therapy* 24.3, pp. 134–140.

Khan, Zia, Christian Hammer, Jonathan Carroll, Flavia Di Nucci, Sergio Ley Acosta, Vidya Maiya, Tushar Bhangale, Julie Hunkapiller, Ira Mellman, Matthew L Albert, et al. (2021). "Genetic variation associated with thyroid autoimmunity shapes the systemic immune response to PD-1 checkpoint blockade". In: *Nature Communications* 12.1, p. 3355.

Kim, Kyung Hwan, Jinhyun Cho, Bo Mi Ku, Jiae Koh, Jong-Mu Sun, Se-Hoon Lee, Jin Seok Ahn, Jaekyung Cheon, Young Joo Min, Su-Hyung Park, et al. (2019). "The First-week Proliferative Response of Peripheral Blood PD-1+ CD8+ T Cells Predicts the Response to Anti-PD-1 Therapy in Solid TumorsPredictive Peripheral Blood Biomarker for Anti-PD-1 Therapy". In: *Clinical Cancer Research* 25.7, pp. 2144–2154. DOI: 10.1158/1078-0432.CCR-18-1449.

Kong, Shengchun, Dominik Heinzmann, Sabine Lauer, and Lu Tian (2022). "Weighted approach for estimating effects in principal strata with missing data for a categorical post-baseline variable in randomized controlled trials". In: *Statistics in Biopharmaceutical Research*, pp. 1–11.

Le, Dung T, Jennifer N Uram, Hao Wang, Bjarne R Bartlett, Holly Kemberling, Aleksandra D Eyring, Andrew D Skora, Brandon S Luber, Nilofer S Azad, Dan Laheru, et al. (2015). "PD-1 blockade in tumors with mismatch-repair deficiency". In: *New England Journal of Medicine* 372.26, pp. 2509–2520. DOI: 10.1056/NEJMoa1500596.

Lipkovich, Ilya, Alex Dmitrienko, and Ralph B D'Agostino Sr (2017). "Tutorial in biostatistics: data-driven subgroup identification and analysis in clinical trials". In: *Statistics in medicine* 36.1, pp. 136–196.

Lu, Steve, Julie E Stein, David L Rimm, Daphne W Wang, J Michael Bell, Douglas B Johnson, Jeffrey A Sosman, Kurt A Schalper, Robert A Anders, Hao Wang, et al. (2019). "Comparison of biomarker modalities for predicting response to PD-1/PD-L1 checkpoint blockade: a systematic review and meta-analysis". In: *JAMA oncology* 5.8, pp. 1195–1204. DOI: 10.1001/jamaoncol.2019.1549.

Mantel, Nathan and David P. Byar (1974). "Evaluation of Response-Time Data Involving Transient States: An Illustration Using Heart-Transplant

Data". In: *Journal of the American Statistical Association* 69.345, pp. 81–86. DOI: 10.1080/01621459.1974.10480131.

McCullagh, Peter and John A Nelder (2019). *Generalized linear models*. Routledge.

Monti, Stefano, Pablo Tamayo, Jill Mesirov, and Todd Golub (2003). "Consensus clustering: a resampling-based method for class discovery and visualization of gene expression microarray data". In: *Machine learning* 52.1, pp. 91–118.

Moons, Karel GM, Patrick Royston, Yvonne Vergouwe, Diederick E Grobbee, and Douglas G Altman (2009). "Prognosis and prognostic research: what, why, and how?" In: *Bmj* 338.

Nardone, Valerio, Rocco Giannicola, Giovanna Bianco, Diana Giannarelli, Paolo Tini, Pierpaolo Pastina, Antonia Consuelo Falzea, Sebastiano Macheda, Michele Caraglia, Amalia Luce, et al. (2021). "Inflammatory markers and procalcitonin predict the outcome of metastatic non-small-cell-lung-cancer patients receiving PD-1/PD-L1 immune-checkpoint blockade". In: *Frontiers in Oncology* 11, p. 684110.

Netterberg, Ida, Chi-Chung Li, Luciana Molinero, Nageshwar Budha, Siddharth Sukumaran, Mark Stroh, E Niclas Jonsson, and Lena E Friberg (2019). "A PK/PD analysis of circulating biomarkers and their relationship to tumor response in atezolizumab-treated non-small cell lung cancer patients". In: *Clinical Pharmacology & Therapeutics* 105.2, pp. 486–495.

Nguyen Duc, Anh, Dominik Heinzmann, Claude Berge, and Marcel Wolbers (2021). "A pragmatic adaptive enrichment design for selecting the right target population for cancer immunotherapies". In: *Pharmaceutical Statistics* 20, pp. 202–211.

Nie, Lei, Eric H. Rubin, Nitin Mehrotra, José Pinheiro, Laura L. Fernandes, Amit Roy, Stuart Bailey, and Dinesh P. de Alwis (2016). "Rendering the 3 + 3 Design to Rest: More Efficient Approaches to Oncology Dose-Finding Trials in the Era of Targeted Therapy". In: *Clinical Cancer Research* 22.11, pp. 2623–2629. ISSN: 1078-0432. DOI: 10.1158/1078-0432.CCR-15-2644.

Ou, Fang-Shu, Stefan Michiels, Yu Shyr, Alex A Adjei, and Ann L Oberg (2021). "Biomarker discovery and validation: statistical considerations". In: *Journal of Thoracic Oncology* 16.4, pp. 537–545.

Overman, Michael J, Ray McDermott, Joseph L Leach, Sara Lonardi, Heinz-Josef Lenz, Michael A Morse, Jayesh Desai, Andrew Hill, Michael Axelson, Rebecca A Moss, et al. (2017). "Nivolumab in patients with metastatic DNA mismatch repair-deficient or microsatellite instability-high colorectal cancer (CheckMate 142): an open-label, multicentre, phase 2 study". In: *The lancet oncology* 18.9, pp. 1182–1191. DOI: 10.1016/S1470-2045(17)30422-9.

Patil, Namrata S, Wei Zou, Simonetta Mocci, Alan Sandler, Marcus Ballinger, Susan Flynn, Marcin Kowanetz, and Priti S Hegde (2021). "C-reactive protein reduction post treatment is associated with improved survival in atezolizumab (anti-PD-L1) treated non-small cell lung cancer patients". In: *PLoS One* 16.2, e0246486.

Polley, Mei-Yin C and James J Dignam (2021). "Statistical considerations in the evaluation of continuous biomarkers". In: *Journal of Nuclear Medicine* 62.5, pp. 605–611.

Powers, Eric, Georgia Sofia Karachaliou, Chester Kao, Michael R Harrison, Christopher J Hoimes, Daniel J George, Andrew J Armstrong, and Tian Zhang (2020). "Novel therapies are changing treatment paradigms in metastatic prostate cancer". In: *Journal of hematology & oncology* 13.1, pp. 1–13. DOI: 10.1186/s13045-020-00978-z.

Putter, H., M. Fiocco, and R. B. Geskus (May 2007). "Tutorial in biostatistics: competing risks and multi-state models". In: *Statistics in Medicine* 26.11, pp. 2389–2430. ISSN: 1097-0258. DOI: 10.1002/sim.2712.

Ribba, Benjamin, Christophe Boetsch, Tapan Nayak, Hans Peter Grimm, Jehad Charo, Stefan Evers, Christian Klein, Jean Tessier, Jean Eric Charoin, Alex Phipps, et al. (2018). "Prediction of the Optimal Dosing Regimen Using a Mathematical Model of Tumor Uptake for Immunocytokine-Based Cancer Immunotherapy Optimal Dosing Regimen for Cancer Immunotherapy". In: *Clinical Cancer Research* 24.14, pp. 3325–3333.

Riley, Richard D, Joie Ensor, Kym IE Snell, Frank E Harrell, Glen P Martin, Johannes B Reitsma, Karel GM Moons, Gary Collins, and Maarten Van Smeden (2020). "Calculating the sample size required for developing a clinical prediction model". In: *Bmj* 368:m441.

Rizopoulos, Dimitris (2012). *Joint models for longitudinal and time-to-event data: With applications in R.* CRC press.

Rizvi, Naiyer A, Matthew D Hellmann, Alexandra Snyder, Pia Kvistborg, Vladimir Makarov, Jonathan J Havel, William Lee, Jianda Yuan, Phillip Wong, Teresa S Ho, et al. (2015). "Mutational landscape determines sensitivity to PD-1 blockade in non–small cell lung cancer". In: *Science* 348.6230, pp. 124–128. DOI: 10.1126/science.aaa1348.

Schandelmaier, Stefan, Matthias Briel, Ravi Varadhan, Christopher H Schmid, Niveditha Devasenapathy, Rodney A Hayward, Joel Gagnier, Michael Borenstein, Geert JMG van der Heijden, Issa J Dahabreh, et al. (2020). "Development of the Instrument to assess the Credibility of Effect Modification Analyses (ICEMAN) in randomized controlled trials and meta-analyses". In: *Cmaj* 192.32, E901–E906.

Seber, George AF and Christopher John Wild (2003). "Nonlinear regression. hoboken". In: *New Jersey: John Wiley & Sons* 62.63, p. 1238.

Silber Baumann, Hanna E., Christophe Boetsch, Jehad Charo, Claire Petry, Volker Teichgräber, and Valerie Cosson (2018). "PKPD analysis of soluble CD25 to characterize the concentration-effect relationship observed

following the administration of Cergutuzumab Amunaleukin, a targeted immunocytokine for cancer immunotherapy".

Smyth, Gordon K (2004). "Linear models and empirical bayes methods for assessing differential expression in microarray experiments". In: *Statistical applications in genetics and molecular biology* 3.1.

Snyder, Alexandra, Vladimir Makarov, Taha Merghoub, Jianda Yuan, Jesse M Zaretsky, Alexis Desrichard, Logan A Walsh, Michael A Postow, Phillip Wong, Teresa S Ho, et al. (2014). "Genetic basis for clinical response to CTLA-4 blockade in melanoma". In: *New England Journal of Medicine* 371.23, pp. 2189–2199. DOI: 10.1056/NEJMoa1406498.

Städler, Nicolas, Frank Dondelinger, Steven M Hill, Rehan Akbani, Yiling Lu, Gordon B Mills, and Sach Mukherjee (2017). "Molecular heterogeneity at the network level: high-dimensional testing, clustering and a TCGA case study". In: *Bioinformatics* 33.18, pp. 2890–2896.

Steyerberg, E.W. (2019). *Clinical Prediction Models: A Practical Approach to Development, Validation, and Updating (second edition).* Springer - Statistics for Biology and Health.

Steyerberg, Ewout W (2018). "Validation in prediction research: the waste by data splitting". In: *Journal of clinical epidemiology* 103, pp. 131–133.

Tanizaki, Junko, Koji Haratani, Hidetoshi Hayashi, Yasutaka Chiba, Yasushi Nakamura, Kimio Yonesaka, Keita Kudo, Hiroyasu Kaneda, Yoshikazu Hasegawa, Kaoru Tanaka, et al. (2018). "Peripheral blood biomarkers associated with clinical outcome in non–small cell lung cancer patients treated with nivolumab". In: *Journal of thoracic oncology* 13.1, pp. 97–105. DOI: 10.1016/j.jtho.2017.10.030.

Tsao, Ming Sound, Keith M Kerr, Mark Kockx, Mary-Beth Beasley, Alain C Borczuk, Johan Botling, Lukas Bubendorf, Lucian Chirieac, Gang Chen, Teh-Ying Chou, et al. (2018). "PD-L1 immunohistochemistry comparability study in real-life clinical samples: results of blueprint phase 2 project". In: *Journal of Thoracic Oncology* 13.9, pp. 1302–1311. DOI: 10.1016/j.jtho.2018.05.013.

Vaddepally, Raju K, Prakash Kharel, Ramesh Pandey, Rohan Garje, and Abhinav B Chandra (2020). "Review of indications of FDA-approved immune checkpoint inhibitors per NCCN guidelines with the level of evidence". In: *Cancers* 12.3, p. 738. DOI: 10.3390/cancers12030738.

Valentinuzzi, Damijan and Robert Jeraj (2020). "Computational modelling of modern cancer immunotherapy". In: *Physics in Medicine & Biology* 65.24, 24TR01.

Van Houwelingen, Hans C. (2007). "Dynamic Prediction by Landmarking in Event History Analysis". In: *Scandinavian Journal of Statistics* 34.1, pp. 70–85. ISSN: 1467-9469. DOI: 10.1111/j.1467-9469.2006.00529.x.

Vega, Diana Merino, Katherine K Nishimura, Névine Zariffa, Jeffrey C Thompson, Antje Hoering, Vanessa Cilento, Adam Rosenthal, Valsamo Anagnostou, Jonathan Baden, Julia A Beaver, et al. (2022). "Changes in

Circulating Tumor DNA Reflect Clinical Benefit Across Multiple Studies of Patients With Non–Small-Cell Lung Cancer Treated With Immune Checkpoint Inhibitors". In: *JCO Precision Oncology* 6, e2100372. DOI: 10.1200/PO.21.00372.

Wages, Nolan A, Cody Chiuzan, and Katherine S Panageas (2018). "Design considerations for early-phase clinical trials of immune-oncology agents". In: *Journal for immunotherapy of cancer* 6.1, pp. 1–10.

Wang, Min, Xiaoyang Zhai, Ji Li, Jingyuan Guan, Shuhui Xu, YuYing Li, and Hui Zhu (2021). "The role of cytokines in predicting the response and adverse events related to immune checkpoint inhibitors". In: *Frontiers in immunology* 12, p. 2894.

Warton, David I and Francis KC Hui (2011). "The arcsine is asinine: the analysis of proportions in ecology". In: *Ecology* 92.1, pp. 3–10.

Wei, Spencer C, Colm R Duffy, and James P Allison (2018). "Fundamental mechanisms of immune checkpoint blockade TherapyFundamental mechanisms of immune checkpoint blockade therapy". In: *Cancer discovery* 8.9, pp. 1069–1086. DOI: 10.1158/2159-8290.CD-18-0367.

Xu, Chenjia, Bin Zhuo, and Hans Erik Rasmussen (2021). "Dose intra-subject escalation to an event (DIETE): A new method for phase 1 dose-finding utilizing systematic intra-subject dose escalation with application to T-cell engagers". In: *Pharmaceutical Statistics* n/a.n/a. DOI: https://doi.org/10.1002/pst.2140.

Zeng, Jing, Xin-Ke Zhang, Hua-Dong Chen, Zhi-Hai Zhong, Qiu-Liang Wu, and Su-Xia Lin (2016). "Expression of programmed cell death-ligand 1 and its correlation with clinical outcomes in gliomas". In: *Oncotarget* 7.8, pp. 8944–8955. DOI: 10.18632/oncotarget.6884.

Zuazo, Miren, Hugo Arasanz, Gonzalo Fernández-Hinojal, Maria Jesus García-Granda, María Gato, Ana Bocanegra, Maite Martínez, Berta Hernández, Lucía Teijeira, Idoia Morilla, et al. (2019). "Functional systemic CD 4 immunity is required for clinical responses to PD-L1/PD-1 blockade therapy". In: *EMBO molecular medicine* 11.7, e10293. DOI: 10.15252/emmm.201910293.
